# The Potential for Treatment Shortening With Higher Rifampicin Doses: Relating Drug Exposure to Treatment Response in Patients With Pulmonary Tuberculosis

**DOI:** 10.1093/cid/ciy026

**Published:** 2018-03-21

**Authors:** Elin M Svensson, Robin J Svensson, Lindsey H M te Brake, Martin J Boeree, Norbert Heinrich, Sarah Konsten, Gavin Churchyard, Rodney Dawson, Andreas H Diacon, Gibson S Kibiki, Lilian T Minja, Nyanda E Ntingiya, Ian Sanne, Stephen H Gillespie, Michael Hoelscher, Patrick P J Phillips, Ulrika S H Simonsson, Rob Aarnoutse

**Affiliations:** 1 Department of Pharmacy, Radboud Institute for Health Sciences, Radboud University Medical Center, Nijmegen, The Netherlands; 2 Department of Pharmaceutical Biosciences, Uppsala University, Sweden; 3 Department of Lung Diseases, Radboud Institute for Health Sciences, Radboud University Medical Center, Nijmegen, the Netherlands; 4 Medical Centre of the University of Munich (LMU), Munich Partner Site, Germany; 5 German Center for Infection Research (DZIF), Munich Partner Site, Germany; 6 The Aurum Institute, Johannesburg, South Africa; 7 School of Public Health, University of Witwatersr, Johannesburg, South Africa; 8 Advancing Treatment and Care for TB and HIV, South African Medical Research Council, Johannesburg, South Africa; 9 University of Cape Town Lung Institute, Cape Town, South Africa; 10 University of Stellenbosch, Cape Town, South Africa; 11 Kilimanjaro Clinical Research Institute, Moshi; 12 Ifakara Health Institute, Bagamoyo; 13 NIMR-Mbeya Medical Research Centre, Mbeya, Tanzania; 14 University of the Witswatersrand, Johannesburg, South Africa; 15 University of St Andrews, United Kingdom; 16 MRC Clinical Trials Unit, University College of London, United Kingdom; 17 Division of Pulmonary and Critical Care Medicine, University of California San Francisco, US

**Keywords:** high-dose rifampicin, pharmacometrics, PK-PD, exposure-response, sputum culture conversion

## Abstract

**Background:**

Tuberculosis remains a huge public health problem and the prolonged treatment duration obstructs effective tuberculosis control. Higher rifampicin doses have been associated with better bactericidal activity, but optimal dosing is uncertain. This analysis aimed to characterize the relationship between rifampicin plasma exposure and treatment response over 6 months in a recent study investigating the potential for treatment shortening with high-dose rifampicin.

**Methods:**

Data were analyzed from 336 patients with pulmonary tuberculosis (97 with pharmacokinetic data) treated with rifampicin doses of 10, 20, or 35 mg/kg. The response measure was time to stable sputum culture conversion (TSCC). We derived individual exposure metrics with a previously developed population pharmacokinetic model of rifampicin. TSCC was modeled using a parametric time-to-event approach, and a sequential exposure-response analysis was performed.

**Results:**

Higher rifampicin exposures increased the probability of early culture conversion. No maximal limit of the effect was detected within the observed range. The expected proportion of patients with stable culture conversion on liquid medium at week 8 was predicted to increase from 39% (95% confidence interval, 37%–41%) to 55% (49%–61%), with the rifampicin area under the curve increasing from 20 to 175 mg/L·h (representative for 10 and 35 mg/kg, respectively). Other predictors of TSCC were baseline bacterial load, proportion of culture results unavailable, and substitution of ethambutol for either moxifloxacin or SQ109.

**Conclusions:**

Increasing rifampicin exposure shortened TSCC, and the effect did not plateau, indicating that doses >35 mg/kg could be yet more effective. Optimizing rifampicin dosage while preventing toxicity is a clinical priority.

## Teaser

We used advanced model-based methods to characterize the relationship between individual rifampicin exposure and antituberculosis treatment response. With data from a trial investigating high-dose rifampicin, a significant relation could be derived, and the clinical impact of increased doses was predicted.

As the leading infectious disease killer, tuberculosis remains a huge public health concern [1]. The efficacy of available tuberculosis therapy is limited, in part, by the long duration of treatment, which is an obstacle to effective tuberculosis control. Effective, safe, and shorter treatment regimens are needed to make meaningful progress toward eliminating tuberculosis globally.

Rifampicin is a key drug in the first-line regimen, and has together with pyrazinamide enabled treatment shortening in the past. The choice of the currently recommended rifampicin dose (10 mg/kg) was not based on optimal efficacy but rather driven by cost and fear of toxic effects [2]. Murine tuberculosis models suggest that the bactericidal and sterilizing effect of rifampicin can be enhanced by increased doses, resulting in significant treatment shortening [3–6]. In patients with tuberculosis, doses up to 40 mg/kg daily were safe and well tolerated over 14 days [7, 8], and a model-based analysis demonstrated that higher rifampicin concentrations could increase the bactericidal activity during the first treatment week significantly [9]. The potential for tuberculosis treatment shortening using high-dose rifampicin was recently studied in a multiarm, multistage trial, also evaluating substitution of ethambutol with either moxifloxacin or SQ109 [10]. Moxifloxacin produces a more rapid initial decline in bacterial load compared to the standard treatment [11]. The candidate drug SQ109 has a novel mechanism of action and is well tolerated [12]. The primary end point was time to stable sputum culture conversion (TSCC), based on liquid cultures until week 12.

A central theorem in clinical pharmacology states that it is the unbound concentration of the active compound(s) at the site of action that drives the effect. Hence, it is of primary interest to describe the relationship between individual drug exposure and response, rather than simply correlate study arm to outcome [10]. Given that exposure observations from the site of action are difficult or impossible to obtain, plasma concentrations are often used as a proxy. Population pharmacokinetic models are powerful tools to capture both the median drug exposure and the variability within a population, and to detect and characterize covariate relationships. Individual exposures can subsequently be linked to models describing the outcome, in this case TSCC. Survival-type outcome data as this can be characterized with time-to-event-analysis using parametric hazard models [13]. Compared with Cox regression [14], well-fitting parametric models are expected to yield higher statistical power and do not necessarily rely on proportionality assumptions [15]. Furthermore, parametric models allow for easy testing of nonlinear covariate-relationships and enable simulations to predict outcomes in different scenarios.

In this article, we describe the population pharmacokinetics of rifampicin, and quantify for the first time the link between rifampicin exposure and treatment response in the multiarm, multistage study, using an innovative model-based approach simultaneously accounting for other predictors. With the developed model, we simulated the expected outcome over a range of rifampicin exposures to assess the potential for treatment shortening.

## METHODS

### Study Design and Data

The study design and enrollment criteria were described in detail in the original publication [10]. In short, this multicenter study included patients newly diagnosed pulmonary tuberculosis from 7 sites in Tanzania and South Africa. Patients were randomized to either the control arm or 1 of 4 experimental arms in a ratio of 2:1:1:1:1; regimens are described in Table 1. Drug concentrations were assessed at day 28 in 20 patients per arm, consecutively enrolled from 2 South African and 2 Tanzanian sites. The morning dose was administered after light breakfast, and blood sampling was conducted within 30 minutes before and 0.5, 1, 2, 3, 4, 6, 8, 12, and 24 hours after the dose. Rifampicin plasma concentrations were determined with a validated ultraperformance liquid chromatography method (accuracy, 95.1%–102.4%; intraday and interday precision, <4.2%l lower limit of quantification, 0.13 mg/L).

**Table 1.** Regimens and Doses for the 5 Study Arms^a^

Arm	Regimen (Once-Daily Administration)^b^				Duration, mo
	Rifampicin Dose, mg/kg	Isoniazid Dose, mg/kg	Pyrazinamide Dose, mg/kg	4th Drug (Dose)	
Control arm	10	5	25^c^	Ethambutol (15–20 mg/kg)^c^	6
Experimental arms					
1	35	5	25	Ethambutol (15–20 mg/kg)	3^**d**^
2	10	5	25	SQ109 (300 mg)	3^**d**^
3	20	5	25	SQ109 (300 mg)	3^**d**^
4	20	5	25	Moxifloxacin (400 mg)	3^**d**^

^a^Pharmacokinetic sampling was conducted in 20 patients per arm.

^b^Adapted in 4 weight bands: 30–37, 38–54, 55–70, and >70 kg.

^c^Pyrazinamide and ethambutol were included only for the first 2 months.

^d^After 3 months with experimental regimens, all patients received another 3 months of standard continuation-phase treatment (rifampicin, 10 mg/kg; isoniazid, 5 mg/kg).

Sputum samples were collected at the start of treatment, weekly until week 8, and again at weeks 10, 12, 14, 17, 22, and 26. Sputum samples were cultured in mycobacterial growth incubator tubes (MGIT960; Becton Dickinson) and on Löwenstein-Jensen medium. TSCC was defined as the time from the start of treatment to the first of 2 consecutive negative cultures.

### Pharmacokinetic Modeling

Nonlinear mixed-effects modeling was used to describe rifampicin pharmacokinetics, estimating both the typical parameter values and the random variability. A published model, developed using data from rifampicin doses of 10–40 mg/kg, was used as a starting point [8]. Given that the pharmacokinetic sampling was conducted 4 weeks after treatment initiation, rifampicin autoinduction was assumed to be completed [8]. Model fit was assessed with goodness-of-fit plots, including visual predictive checks, and the Akaike information criterion. The investigated covariates were body weight, fat-free mass [16], sex, country, human immunodeficiency virus status, and the presence of lung cavitation. The decision on inclusion in the final model was based on statistical significance (*P* < .05) and biological plausibility. Results were compared with those of a previously published noncompartmental analysis (NCA) [10].

### Pharmacodynamic Modeling

The response variable was TSCC, derived separately based on liquid and solid culture results. Both variables were modeled using a time-to-event approach, but the liquid culture TSCC was the primary focus. Different parametric hazard distributions determining TSCC were evaluated, including constant, Weibull, and surge functions. In the selected base model, the covariates evaluated included baseline bacterial load (time to positivity in liquid medium), sex, body weight, country, study site, and lung damage (radiographic scoring and presence of cavitation [17]). Because TSCC may be affected by missing or contaminated sputum samples, a metric to account for this (percentage of unavailable culture results until last planned visit or before dropout) was therefore derived and evaluated. The same criteria for covariate inclusion were applied as for the pharmacokinetic analysis, and the same methods of evaluating model fit.

### Exposure-Response Analysis

Treatment-related covariates were investigated in a sequential exposure-response analysis. The influence of rifampicin exposure in plasma (area under the curve from 0 to 24 hours after dose [AUC_0–24h_], or maximal concentration [*C*_max_] derived using the final population pharmacokinetic model) was tested using linear, power, and maximal effect (*E*_max_) models. In addition, the choice of fourth drug (ethambutol, moxifloxacin, or SQ109) and the influence of pyrazinamide and isoniazid exposure (published NCA-derived AUC_0–24h_ or *C*_max_ [10]) were investigated.

Because pharmacokinetic information was obtained in a subset of the patients, individual exposure metrics were missing for about 75% of the patients. To enable analysis of the whole population and use information on rifampicin dose and demographics, we applied imputation methods. Individual rifampicin exposures were predicted using the developed population pharmacokinetic model, the administered dose, and relevant patient characteristics. Adequacy of the imputation was evaluated in the patients with pharmacokinetic data available by comparing imputed and observed metrics. Selection of parameter-covariate relationships were performed with single imputation, and all included covariates were reevaluated for statistical significance with backward deletion. The final parameter values and their uncertainty were derived after multiple imputation (n = 100) using Rubin’s rules [18, 19]. For pyrazinamide and isoniazid, the pharmacokinetic metrics were imputed to the median per absolute dose of the corresponding NCA-derived variable in the subset of patients with pharmacokinetic observations.

### Simulations of Clinical Impact

To evaluate the impact of included covariates, secondary outcome metrics, such as median TSCC and conversion rates at week 8 and 12, were simulated under a number of scenarios, varying a single covariate at a time. A data set with 10000 virtual tuberculosis patients formed the basis for the simulations. The population was created by sampling from covariate distributions mimicking those observed in the study (see Supplementary Material).

### Software

All software used is described in the Supplementary Material.

## RESULTS

### Patients and Data

In total, 365 patients were included in the study. Two patients with drug resistance were excluded, leaving 363 patients in this analysis. Of the 100 patients planned for pharmacokinetic measurements, rifampicin results were available for 97. Detailed demographics of the population have been described elsewhere [10]; patient characteristics important to our analyses are listed in Table 2.

**Table 2.** Patient Characteristics Important for Pharmacokinetic and Pharmacodynamic Modeling

Patient Characteristic	Population Included in Analysis	
	PK Analysis (n = 97)	PD Analysis (n = 363)
Age, median (range), y	34 (18–56)	33 (18–86)
Weight, median (range), kg	54 (35–80)	53 (35–86)
Fat-free mass, median (range), kg^a^	44.7 (28.8–55.9)^b^	44.6 (28.8–61.0)^b^
Female sex, No. (%)	29 (30)	107 (29)
HIV infection, No. (%)	2 (2)	24 (7)
Time to positivity in mycobacterial growth incubator tube at baseline, median (range), d^c^	4.3 (0.54–17.1)^d^	4.4 (0.54–21.7)^e^
Lung cavitation present at baseline, No. (%)	50 (76)^f^	176 (70)^g^
Radiographic score at baseline, median (range)^h^	60 (3–113)^f^	58 (3–113)^g^

Abbreviations: HIV, human immunodeficiency virus; PD, pharmacodynamic; PK, pharmacokinetic.

^a^Evaluated only in the PK model and calculated from body weight, height, and sex according to the formula described by Janmahasatian et al [16].

^b^Data missing in 1 patient.

^c^Evaluated only in the PD model.

^d^Data missing in 2 patients.

^e^Data missing in 6 patients.

^f^Data missing in 31 patients.

^g^Data missing in 111 patients.

^h^Defined as percentage of lung affected plus 40 if cavitation is present, as described by Ralph et al [17].

From the 97 patients, there were in total 956 measurements of rifampicin plasma concentrations available. Predose samples were excluded from the model fitting owing to missing dose history, as were 24 samples with suspected erroneous time recordings, resulting in 845 observations, of which 654 were above the limit of quantification.

Of the 363 patients included in the pharmacodynamic analysis, 296 reached stable culture conversion within 26 weeks. Among the patients not reaching conversion, 32 of 67 patients dropped out before the end of the study or lacked a culture result from the week 26 visit and were censored in the time-to-event analysis at the time of their last recorded culture result. For liquid cultures, the median proportion of results unavailable per patient was 17% (range, 0%–76%), and the proportions differed substantially between study sites.

### Pharmacokinetic Modeling

**Figure 1.**
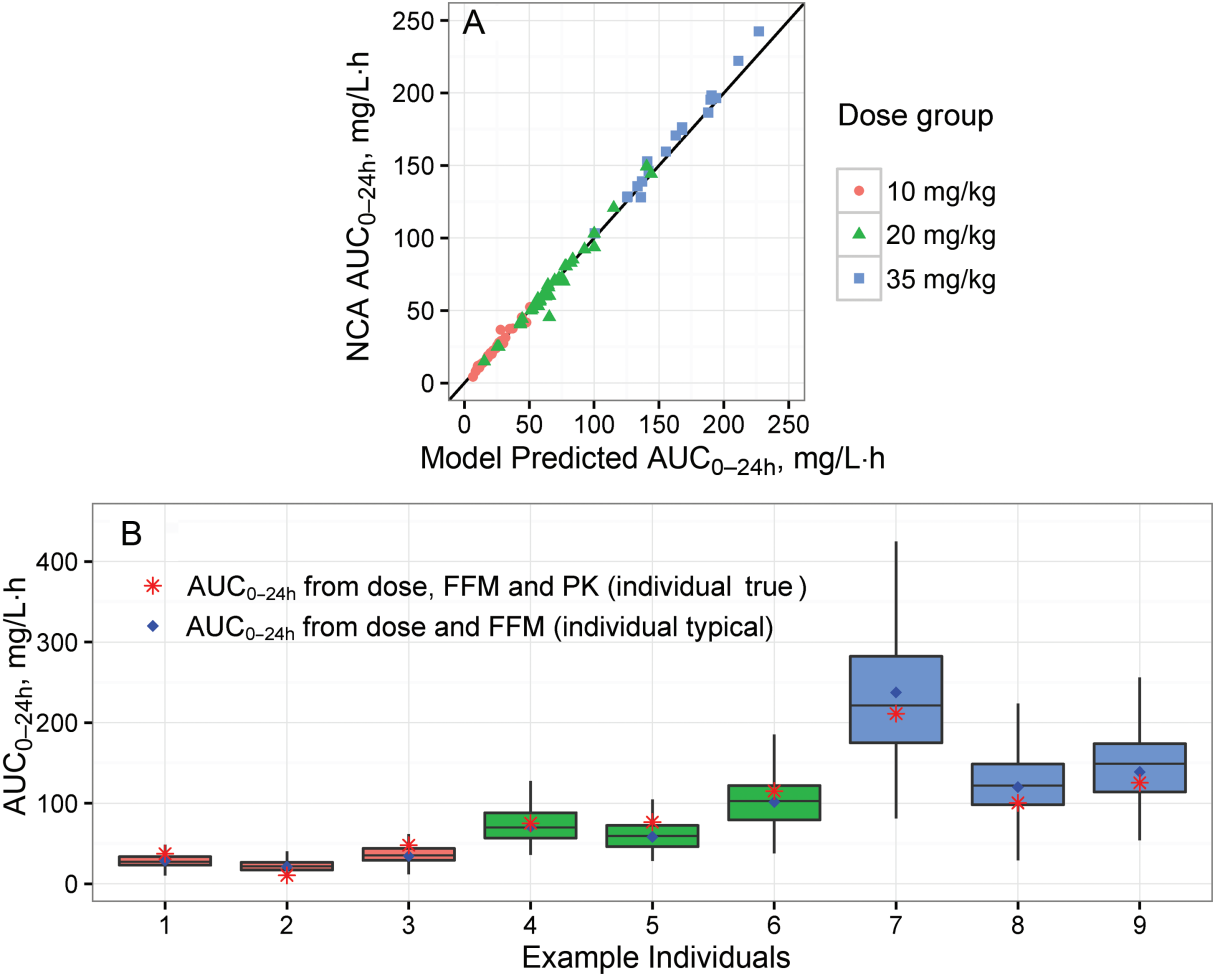
Evaluation of the pharmacokinetic model. *A,* Comparison between individual rifampicin area under the concentrations curve from 0 to 24 hours after the dose (AUC_0–24h_) at day 28 derived by noncompartmental analysis (NCA) and by the model, per rifampicin dose level (circles represent 10 mg/kg; triangles, 20 mg/kg; squares, 35 mg/kg). *B,* Distribution of AUC_0–24h_ values (box-and-whisker plots) simulated including interindividual variability for 9 representative patients (dose level 10 mg/kg for individuals 1–3, 20 mg/kg for individuals 4–6, and 35 mg/kg for individuals 7–9) with corresponding individual typical AUC_0–24h_, that is, expected value given the individual’s dose and fat-free mass (FFM), and “true” AUC_0–24h_, that is, expected value given the individual’s rifampicin dose, FFM and observed rifampicin plasma concentrations. The 9 patients were chosen to cover the 3 most common absolute rifampicin doses per dose level, simply selecting the first included patient per dose. Boxes represents the first, second, and third quartiles, and whiskers extend to the highest and lowest values within 1.5 times the interquartile range. Abbreviation: PK, pharmacokinetics.

The previously developed population pharmacokinetic model fitted the data adequately. Details on model structure, modifications from the original, parameter estimates and model evaluation are presented in the Supplementary Material and Figure S1. Evaluations showed that the model could well predict individual exposures (AUC_0–24h_) derived from NCA (Figures 1A and S2) and generated plausible individual exposure distributions for the multiple imputation procedure (Figure 1B).

### Pharmacodynamic Modeling

The best base hazard model for TSCC derived from liquid cultures was a surge function defined by amplitude, peak time, and peak width (Akaike information criterion, 3053; compared with 3231 and 3162 for constant and Weibull hazard, respectively; see also Supplementary Figure S3). The covariates found to be statistically significant were baseline bacterial load and the percentage of unavailable culture results. The latter explained a marked difference in TSCC between study sites, caused by varying levels of missing data. Further details and results for TSCC derived from solid cultures are described in the Supplementary Material.

### Exposure-Response Analysis

Individual rifampicin exposures were found to significantly affect the hazard (AUC_0–24h_ to a greater extent than *C*_max_), with a high exposure leading to an increased probability of early sputum culture conversion (SCC). A sigmoid relationship did not describe the data better than a linear relationship; therefore, a maximal effect could not be reliably estimated. The choice of fourth drug with the applied dose had an additional statistically significant effect, with moxifloxacin decreasing TSCC and SQ109 increasing TSCC compared with ethambutol. An effect of pyrazinamide exposure (AUC_0–24h_ or *C*_max_) was only statistically significant in univariate analysis, and this was not included in the final model. No effect of isoniazid exposure was detected.

**Figure 2.**
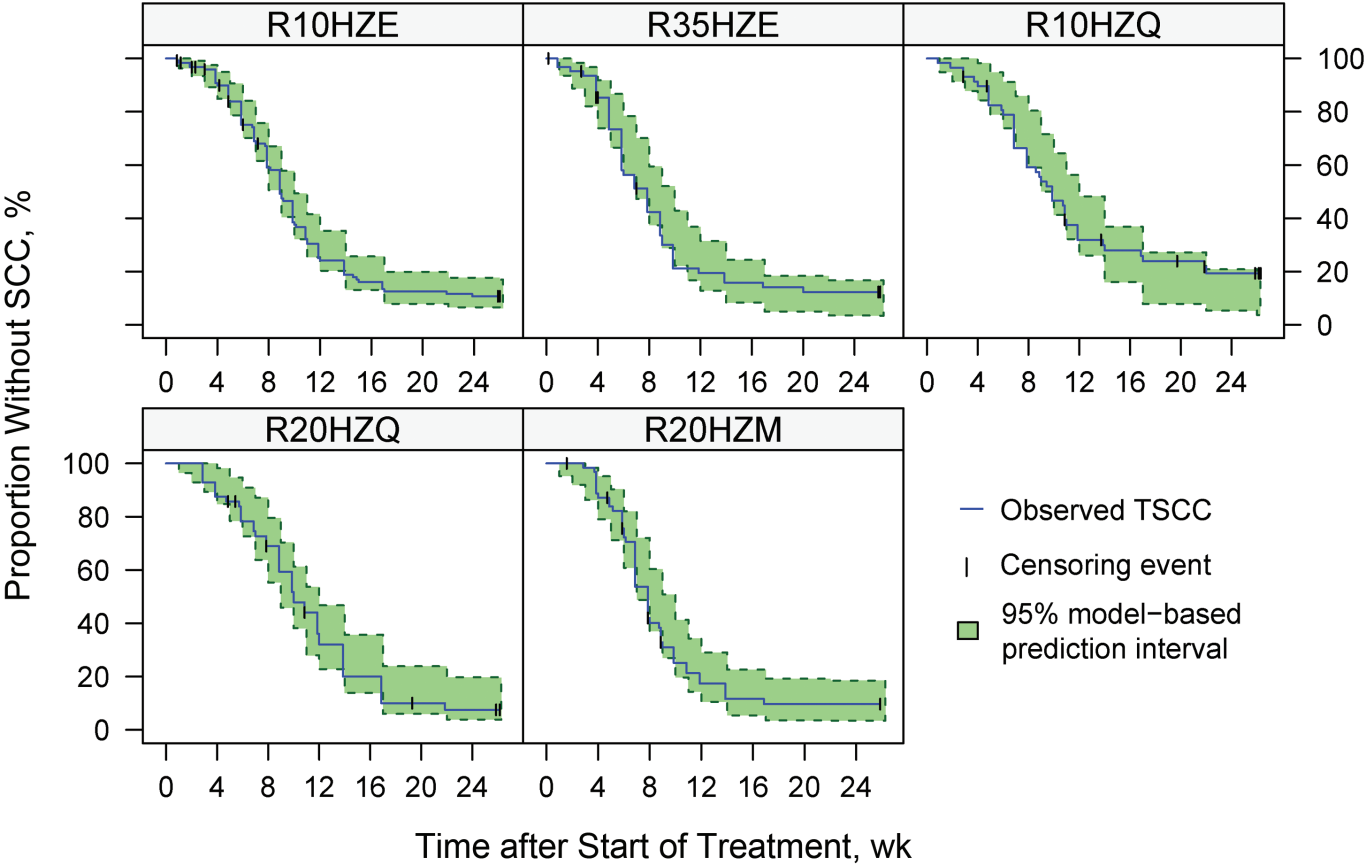
Evaluation of the final time-to-event model describing time to sputum culture conversion (TSCC) based on liquid cultures, per study arm, in the form of a visual predictive check. Solid lines represent Kaplan-Meier curves based on the observed data. Vertical tick marks signify censored data, and shaded area outlines 95% prediction interval based on simulations using the model. Study arms include the control arms (R10HZE) and experimental arms 1–4 (R35HZE, R10HZQ, R20HZQ and, R20HZM). (Number in arm name represent rifampicin dose [in milligrams per kilogram] used in the first treatment period, 2 months for R10HZE and 3 months for all other arms). Abbreviation: SCC, sputum culture conversion.

The detailed parameterization of the covariate-parameter relationships, statistical significance for each included covariate, final parameter values, and the model code are included in the Supplementary Material. The final model’s good description of the observed data is demonstrated in Figure 2. The hazard-function per study arm is shown in Supplementary Figure S4. Results for TSCC derived from solid cultures were similar (see Supplementary Material).

### Simulations of Clinical Impact

**Figure 3.**
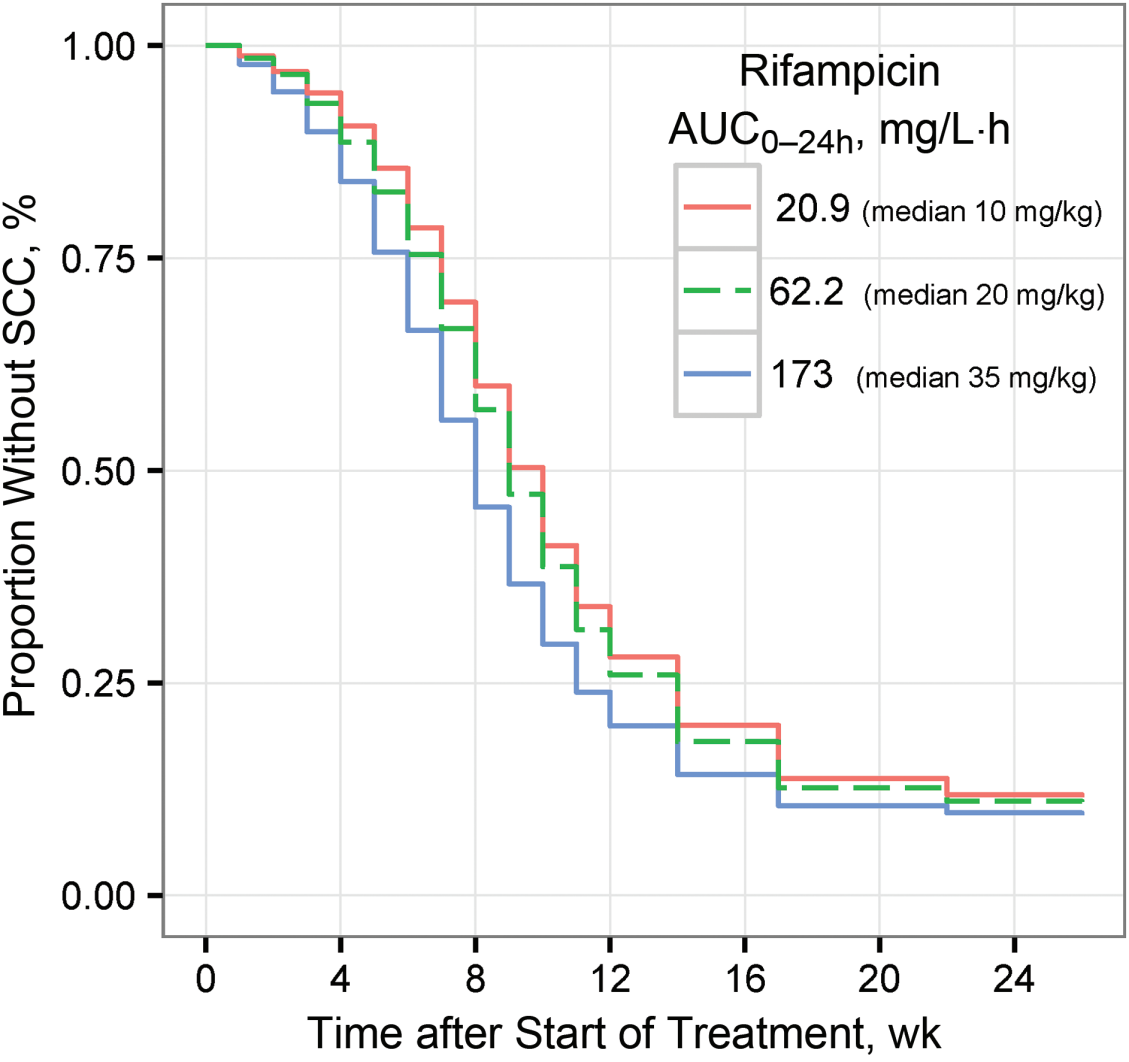
Predicted proportion without sputum culture conversion (SCC) versus time after start of treatment for virtual populations of patients (n = 10000) having rifampicin area under the concentration versus time curve from 0 to 24 hours after the dose (AUC_0-24h_) of either 21, 62, or 173 mg/L·h (median observed exposure in dose groups 10, 20 and 35 mg/kg, respectively), and standard doses of isoniazid, pyrazinamide, and ethambutol. The distribution of baseline bacterial load and missing sputum samples in the simulation mimicked the results in the study used to build the models.

**Figure 4.**
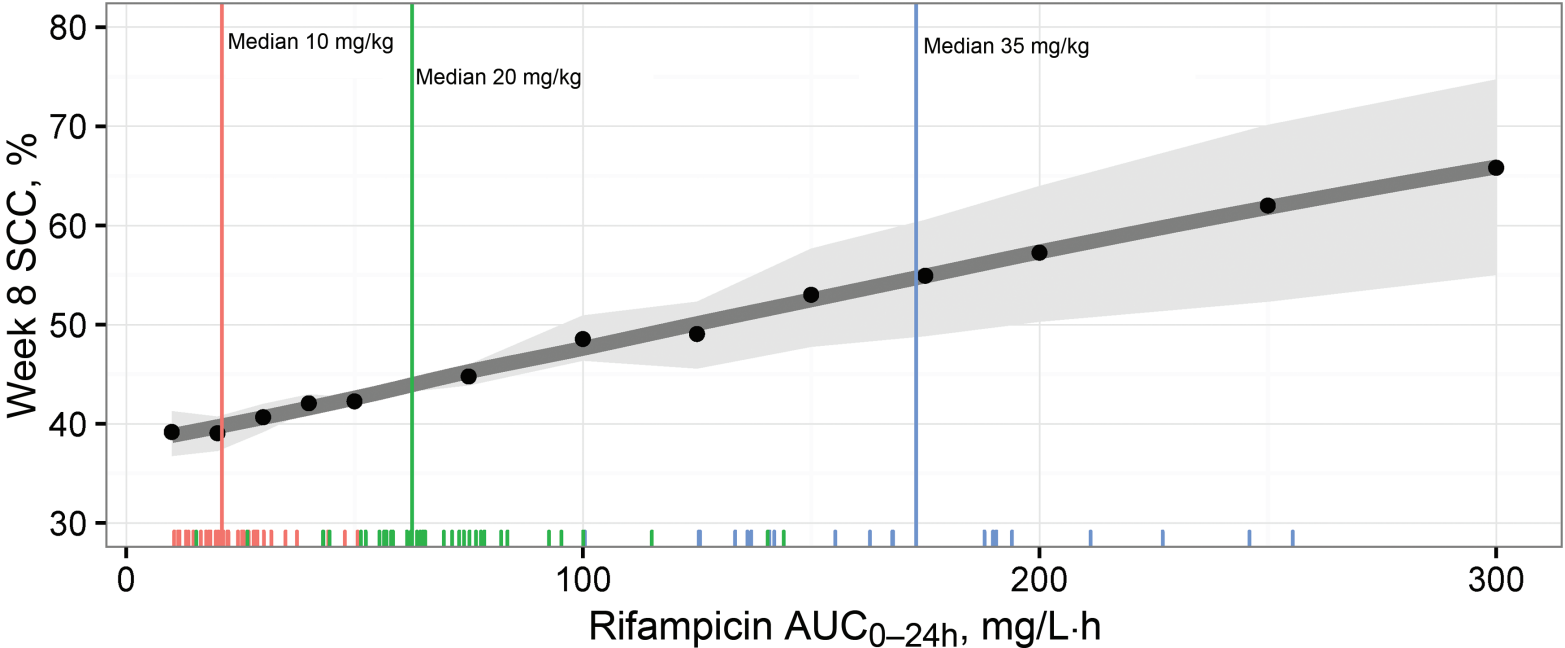
Expected proportion of patients with sputum culture conversion (SCC) at week 8 over varying rifampicin exposures for a virtual population of patients (distribution of baseline bacterial load and missing sputum samples mimicking that of the study) treated with standard doses of isoniazid, pyrazinamide, and ethambutol. Black dots represents simulation results (n = 10000 in each); dark gray line, a locally weighted smooth of the simulation results; and light gray shaded area, 95% confidence interval based on the uncertainty in the estimate of the parameter for rifampicin effect. Vertical lines represent median observed exposures in the dose groups 10 (*red*), 20 (*green*), and 35 (*blue*) mg/kg, respectively, and tick marks at the bottom of the graph are individual observed exposures. Abbreviation: AUC_0–24h_, area under the curve from 0 to 24 hours after dose.

TSCC was shortened with increased rifampicin exposure, as demonstrated in Figure 3, showing the expected TSCC profiles in a virtual population having rifampicin exposure equal to the median exposures observed in the 10-, 20-, and 35-mg/kg groups. Figure 4 shows the expected increase in proportion of patients with SCC at week 8, with increasing rifampicin exposures for the same virtual population, for example, from 39% (95% confidence interval, 37%–41%) to 55% (49%–61%), with rifampicin AUC_0–24h_ increasing from 20 to 175 mg/L·h (representative of 10 and 35 mg/kg, respectively). For a population without any missing culture results, the corresponding values would range from 54% (95% confidence interval, 52%–56%) to 72% (65%–78%) (Supplementary Figure S5). The substantial shortening effect on TSCC caused by low baseline bacterial load is depicted in Supplementary Figure S6, and Supplementary Figure S7 shows the impact of substituting ethambutol for either SQ109 or moxifloxacin.

## DISCUSSION

In this pioneering analysis we linked individual plasma exposures of the key drug rifampicin to TSCC, a commonly used end point in phase II tuberculosis studies. Higher rifampicin exposure during the first 3 months of treatment was shown to shorten TSCC for patients with pulmonary tuberculosis. The relationship between rifampicin exposure and TSCC was statistically significant for both liquid and solid cultures. No maximal effect of rifampicin could be derived within the observed range, indicating that doses even higher than 35 mg/kg may yield additional benefit, if they can be safely and practically administered. However, the modest magnitude of the impact on SCC predicted for very high rifampicin exposures (Figure 4 and Supplementary Figure S3) suggests that increased rifampicin doses may on its own be insufficient for shortening treatment more than to 4 months.

The original analysis of this study only included between-regimen comparisons [10], whereas our model-based analysis could also detect the impact of individual compounds. Patients receiving SQ109 instead of ethambutol actually had a lower probability of culture conversion, predicted to delay SCC 1 week for a typical patient given 10 mg/kg of rifampicin. It remains unclear whether SQ109 exposure levels were sufficiently high (pharmacokinetic data not yet available); a potential interaction with rifampicin may have affected exposure levels. However, we infer that it is unlikely that SQ109 applied in the studied dose could play a role in shortening of tuberculosis treatment. Substitution with moxifloxacin was predicted to typically generate a 1-week-shorter TSCC. This agrees with previous results [11, 20, 21].

The large effect of baseline bacterial load has been documented in previous studies [22–24], and it is physiologically plausible that a larger bacterial burden takes longer to be cleared. The impact of missing culture results was also expected. Missing culture results due to unavailable samples or contamination will intrinsically delay conversion and must therefore be taken into account in any analysis. The pragmatic approach taken here was to introduce a covariate quantifying the proportion of missing culture results per patient. Better still than accounting for missing culture results would be to avoid having them, for example, by performing multiple cultures per time point [25], or by using novel methods to quantify mycobacteria, such as the molecular bacterial load assay [26]. This assay is a polymerase chain reaction–based method and as such is not sensitive to contamination. It generates results faster, beneficial especially for adaptive study designs, but it has not been evaluated against clinical outcomes to date.

In other work based partly on the same data, higher pyrazinamide exposure has been suggested to shorten TSCC [27]. In our analysis, individual pyrazinamide exposure was a statistically significant predictor for TSCC only when baseline bacterial load was not controlled for. A limitation was the simplistic imputation of exposure in patients lacking pharmacokinetic data based on the typical observed pyrazinamide exposure per dose. However, given the modest interindividual variability in pyrazinamide pharmacokinetics [28], the imputation is expected to perform reasonably well. No link between isoniazid exposure and TSCC could be identified. The lack of a statistically significant relationship should not be interpreted as a demonstration of that higher exposures do not yield additional benefits; it may instead be an effect of limited power.

Time-to-event analysis with parametric hazard models has several advantages over classic Cox regression, and it has recently been used in a similar analysis focused on rifapentine [29]. The constant proportionality of hazards, a necessary condition in Cox regression, is difficult to fulfill for complex pharmacological exposure-response relationships and time-varying effects [30]. The statistical power is generally higher with a parametric hazard model than with nonparametric approaches [31]. Furthermore, the developed model can be used to predict outcomes of future trials, something that is imperative in the decision-making process moving novel antituberculosis regimens forward, and for clinical trials simulations optimizing study designs. On the other hand, parametric models are better only when they describe the true underlying hazard distribution well, which we have ensured with stringent model evaluation.

The current analysis has some limitations. The evaluated outcome parameter, TSCC, is an intermediate marker substituting for the actual clinical end point in tuberculosis treatment, which is relapse-free cure. Although TSCC and month 2 SCC have been used as primary end points in recent phase II trials leading to accelerated and conditional regulatory approval [32, 33] and have been linked to relapse rate [34], there is no clear evidence of the predictive performance of such metrics in correlating with the clinical end point [35].

The phase III study of shorter moxifloxacin-containing regimens failed to demonstrate noninferiority for relapse-free cure compared to standard of care, even though the median TSCC was significantly shorter in the experimental arms [11]. A potential reason for the lack of sterilizing activity of the moxifloxacin-containing regimens may be the inability of this drug to penetrate into tuberculosis lesions where persistent mycobacteria can reside [36]. Rifampicin, on the other hand, penetrates well into tuberculosis lesions and is known to be effective against persistent and intracellular bacteria and to drive prevention of relapse in animal models [5, 37–39]. Therefore, we postulate that the shorter TSCC seen with higher rifampicin exposure translates to a higher frequency of relapse-free cure, but this must be confirmed in clinical trials. The scope of this analysis did not include an exposure-toxicity component; earlier work found no link between rifampicin dose and adverse events [7, 10].

Limitations of the final model’s simulation properties originate from the nonlinear relationships in the pharmacokinetic model and the linear exposure-response relation without a maximum effect. Both properties make simulations of doses and exposures far outside the observed range more uncertain. Furthermore, only just over a quarter of the patients had individual rifampicin concentrations observed. This shortcoming was partly mitigated by application of a multiple imputation procedure, enabling us to keep all patients with outcome data in the analysis and expected to provide better estimate of parameter uncertainty than single imputation [40]. Finally, the pharmacokinetic model was validated only internally, no free rifampicin concentrations were measured, and the potential contribution of rifampicin’s less active 25-desacetyl metabolite was not considered.

In conclusion, with a model-based exposure-response analysis, we were able to link increasing rifampicin exposure to modestly shorter TSCC. A crucial new finding was that maximal effect of rifampicin was not reached, indicating that the optimal dose from an efficacy standpoint could be higher than 35 mg/kg. A safety assessment of 50 mg/kg is already ongoing in the extension of the HIGHRIF1 study [7]. Presupposing new tablet formulations to manage pill burden, we suggest that high-dose rifampicin could be an important component of a shortened tuberculosis regimen, simplifying therapy for patients and management of treatment programs. The most appropriate rifampicin dose, balancing efficacy and safety aspects, remains to be defined and should be further studied in well-designed and sufficiently powered clinical trials.

## Supplementary Data

Supplementary materials are available at *Clinical Infectious Diseases* online. Consisting of data provided by the authors to benefit the reader, the posted materials are not copyedited and are the sole responsibility of the authors, so questions or comments should be addressed to the corresponding author.

Supplementary Material

## Notes

***Acknowledgments.*** We thank the patients and site staff participating in the original study, without whom none of this work would have been possible. We also thank Arthur Pistorius, Center for Molecular and Biomolecular Informatics, Radboudumc, for assisting with the computational infrastructure, and David Burger, Department of Pharmacy, Radboudumc, for providing the research environment that made our pharmacometric analysis possible.

***Financial support.*** This work was supported by the European and Developing Countries Clinical Trials partnership (grants IP.2007.32011.011, IP.2007.32011.012, and IP.2007.32011.013) and the German Ministry for Education and Research (grant 01KA0901). The original study conducted within the PanACEA consortium.

***Potential conflicts of interest.*** All authors: No reported conflicts of interest. All authors have submitted the ICMJE Form for Disclosure of Potential Conflicts of Interest. Conflicts that the editors consider relevant to the content of the manuscript have been disclosed.
