# Supplementary material for: The Potential for Treatment Shortening With Higher Rifampicin Doses: Relating Drug Exposure to Treatment Response in Patients With Pulmonary Tuberculosis
Source: Clin Infect Dis. 2018 Mar 21;67(1):34–41. doi: 10.1093/cid/ciy026 (PMC6005123; doi:10.1093/cid/ciy026)
Supplement: Supplementary Material [file ciy026_suppl_supplementary_material.pdf]

Supplementary material

The potential for treatment shortening with higher rifampicin doses: relating drug exposure to treatment response in patients with pulmonary tuberculosis

Elin M Svensson[1,2], Robin J Svensson[2], Lindsey HM te Brake[1], Martin J Boeree[1], Norbert Heinrich[3,4], Sarah Konsten[3,4], Gavin Churchyard[5,6,7], Rodney Dawson[8], Andreas H Diacon[9] , Gibson S Kibiki[10], Lilian T Minja[11], Nyanda E Ntingiya[12], Ian Sanne[13], Stephen H Gillespie[13], Michael Hoelscher[3,4], Patrick PJ Phillips[15,16], Ulrika SH Simonsson[2], Rob Aarnoutse[1]

1. Department of Pharmacy, Radboud Institute for Health Sciences, Radboud university medical center, Nijmegen, The Netherlands
2. Department of Pharmaceutical Biosciences, Uppsala University, Uppsala, Sweden
3. Medical Centre of the University of Munich (LMU), Munich, Germany
4. German Center for Infection Research (DZIF), Munich Partner Site, Munich, Germany
5. The Aurum Institute, Johannesburg, South Africa
6. School of Public Health, University of Witwatersrand, Johannesburg, South Africa
7. Advancing Treatment and Care for TB and HIV, South African Medical Research Council, Johannesburg, South Africa.
8. University of Cape Town Lung Institute, Cape Town, South Africa
9. University of Stellenbosch, Cape Town, South Africa
10. Kilimanjaro Clinical Research Institute, Moshi, Tanzania
11. Ifakara Health Institute, Bagamoyo, Tanzania
12. NIMR-Mbeya Medical Research Centre, Mbeya, Tanzania
13. University of the Witswatersrand, Johannesburg, South Africa
14. University of St Andrews, St Andrews, United Kingdom
15. MRC Clinical Trials Unit, University College of London, London, United Kingdom
16. Division of Pulmonary and Critical Care Medicine, University of California San Francisco, San Francisco, United States of America

Contents

Software 3
Population for simulations of clinical impact 3
Pharmacokinetic modeling 4
Parameter estimates 5
Model evaluation 6
Histogram of exposures 7
Results TSCC liquid cultures 8
Results TSCC solid cultures 12
NONMEM control streams 13
Final model TSCC from liquid cultures 13
Final model TSCC from solid cultures 15

Software

Data management, post processing of results and plotting were performed in R (R Foundation for Statistical Computing, Vienna, Austria), partially using the Xpose package (Department of Pharmaceutical Biosciences, Uppsala University, Uppsala, Sweden). The modeling and simulations were performed in NONMEM 7.3 (Icon Development Solutions, Ellicott City, Maryland, USA), aided by PsN (Department of Pharmaceutical Biosciences, Uppsala University, Uppsala, Sweden) and Pirana (Pirana Software & Consulting, San Francisco, USA).

Population for simulations of clinical impact

The virtual population (n=10000) used as the basis for the simulations of clinical impact was created by sampling from parametric covariate distributions mimicking the distributions observed in the study data. The two non-treatment related covariates included in the final model was baseline bacterial load and proportion of missing samples. For baseline bacterial load a Box-Cox transformed normal distribution (SD 0.35, Box-Cox parameter 0.7) around the median observed time to positivity (4.37 days) was used. If a simulated value was higher than 42 days it was truncated to 42 days. For the proportion of missing sample results, a three-level uniform distribution was used: 59% of the subjects missing between 0 and 20% of the planned samples, 28% missing between 20 and 40% and 13% missing between 40 and 60%. For comparison, simulations were also conducted assuming 0% missing sample results for all subjects. The same sampling schedule as in the original study was implemented and all subjects were assumed to remain in the study until week 26 (no dropout).

Pharmacokinetic modeling

The previously developed population PK model generally fitted the data well. The model includes a single distribution compartment, absorption through a dynamic transit compartment model, and a Michelis-Menten function limiting the clearance at high concentrations. The previously described nonlinear increase in bioavailability with higher doses was not supported in this dataset and therefore simplified to a linear relationship. The relative bioavailability was fixed to 1 for a dose of 450 mg, thereafter increasing with the estimated slope coefficient. Further modifications to the original model included a reduction of the number of random effects, and an addition of a correlation between inter-individual variability in mean transit time and bioavailability. Samples below the limit of quantification (BLQ) were excluded in the estimation step. The model's ability to adequately predict BLQ samples was assessed by evaluating the full dataset including BLQ samples using the final estimated parameters and the M3 method (i.e. quantifying probability of a sample being BLQ).

Allometric scaling with fat-free mass as size descriptor, and coefficients fixed to the expected values (0.75 and 1 for clearance and volume of distribution, respectively) were included. Fat-free mass performed slightly better than total body weight. Gender and presence of lung cavitation did not have a statistically significant impact on clearance or volume of distribution. HIV-infection could not be evaluated due to the low number of HIV-positive patients included. There was a statistically significant but small effect of country on volume of distribution (13% lower in South Africa than Tanzania). Given the lack of a scientific rational and clinical significance, this effect was not included in the final model.

The final parameter estimates with their uncertainty are reported in Table S1. The good fit of the model to the observed concentrations and the proportion BLQ samples is demonstrated in the visual predictive check in Figure S1. The NONMEM code detailing the parameterization is included last in this supplementary material.

Parameter estimates

Table S1. Maximum likelihood estimates of parameter values from the final model including uncertainty determined by the covariance step implemented in NONMEM. The typical values of the maximal elimination rate and the volume of distribution are representative for a patient with fat-free mass of 44.6 kg.

Parameter	Estimate	Relative standard error [%]
Fixed effects		
V_{max} [mg/h/44.6kg]	339	13.1
k_m [mg/L]	11.7	18.3
Volume of distribution [L/44.6kg]	56.5	3.9
Absorption rate constant [h^{-1}]	1.01	13.6
Mean transit time [h]	1.15	11.7
Number of transit compartments	4.99	21.8
Slope linear increase in F [%/1000mg]	13.5	45.5
Random effects (CV or correlation) [%]		
IIV in k_m	32.6	12.0
IIV in absorption rate constant	56.7	19.0
IIV in mean transit time	81.9	10.5
Correlation mean transit time and bioavailability	-70.8	*
IIV in bioavailability	16.1	15.7
IIV in number of transit compartments	137	12.3
Residual error		
Proportional [%]	16.2	4.1
Additive [mg/L]	0.0361	13.7

Abbreviations: V_{max}, maximal elimination rate; k_m, rifampicin concentration at which the elimination is half-maximal; IIV, inter-individual variability; CV, coefficient of variance

* Not calculated, relative standard error of corresponding covariance estimate was 32.8%

Model evaluation

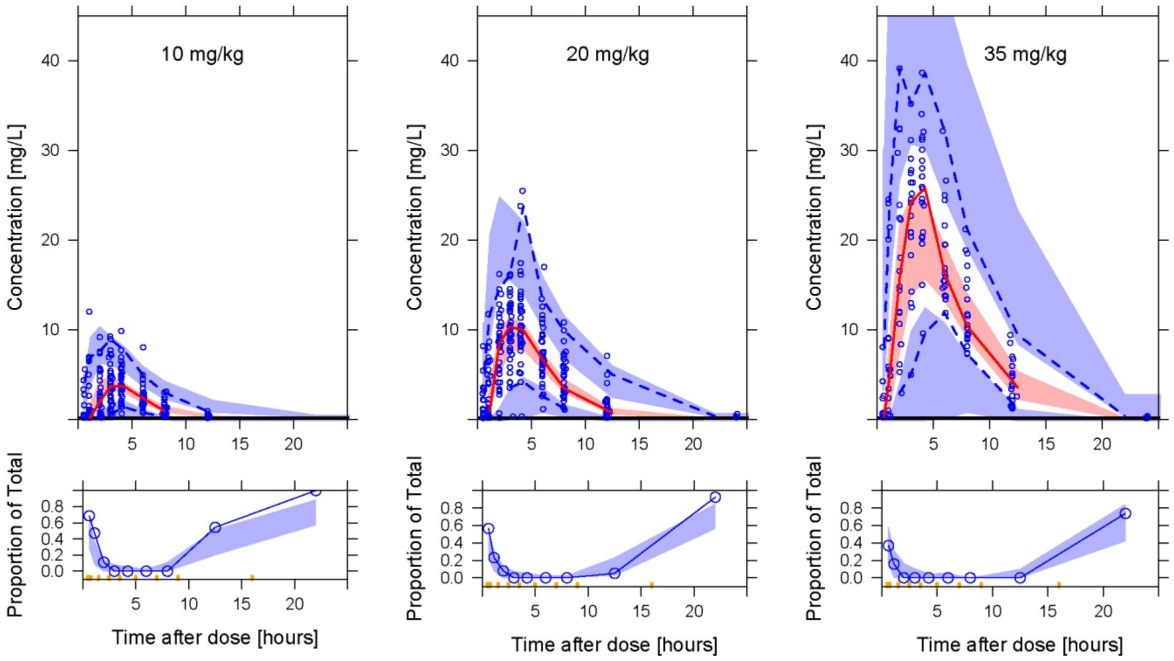

Figure S1. Visual predictive check of observed concentrations (upper panels) and the proportion of samples below the limit of quantification (lower panels), per dose group. In the upper panels blue rings represent the observed rifampicin concentrations, the lines represent the 2.5th, 50th and 97.5th percentiles of the observed data, and the shaded areas are the 95% confidence intervals of the same percentiles based on data simulated by the final model. In the lower panels the blue rings represent the observed proportions samples below the limit of quantification per bin (indicated by the yellow tick marks), and the shaded area represents the 95% confidence intervals of the same proportions based on data simulated by the final model.

Histogram of exposures

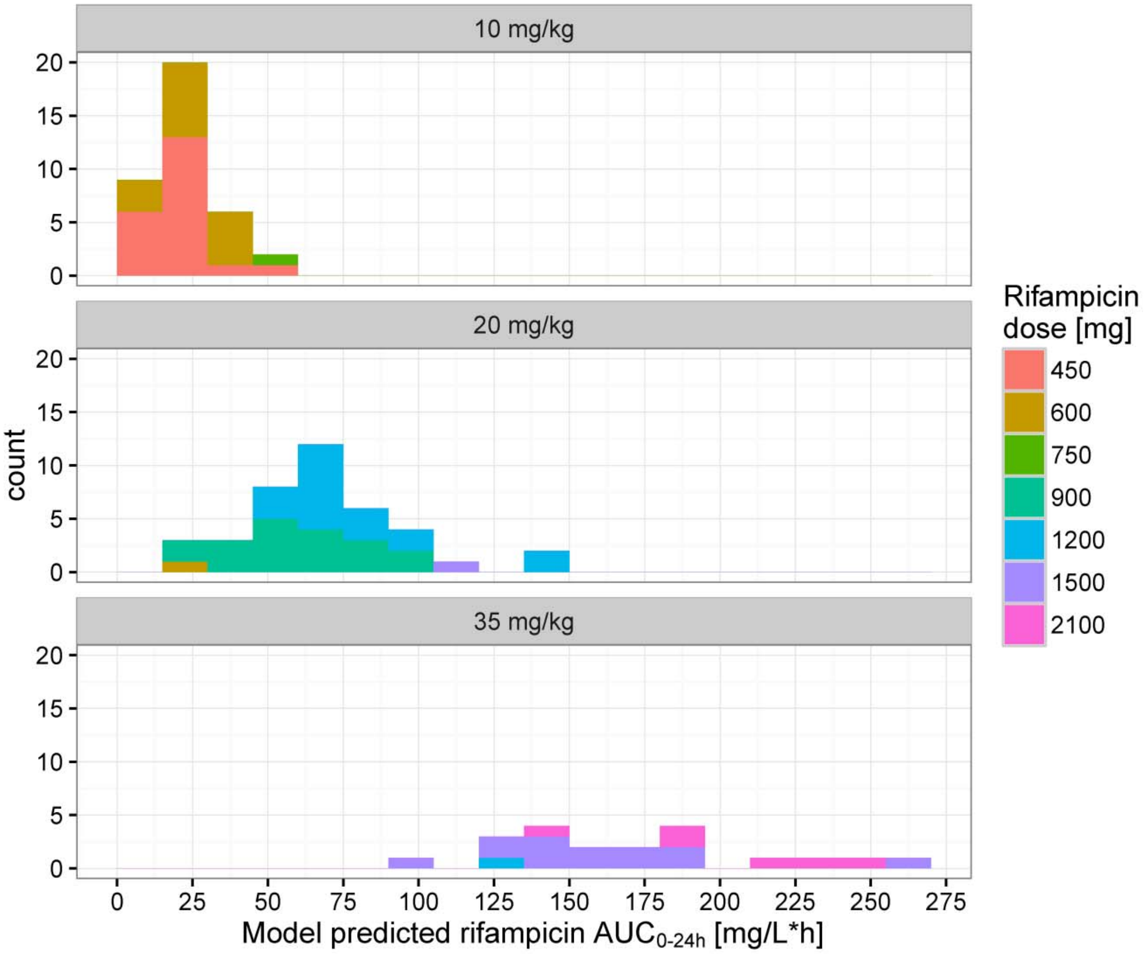

Figure S2. Histogram of individual model predicted rifampicin $AUC_{0\text{-}24h}$ at day 28 plotted in panels per dose level (10, 20 and 35 mg/kg) and colored according to absolute rifampicin dose (mg).

Results TSCC liquid cultures

The goodness of fit for different base hazard models is shown in Figure S3, comparing two standard distributions (constant and Weibul hazard) to the selected surge function. The poor fit of the standard models to the observed data is apparent.

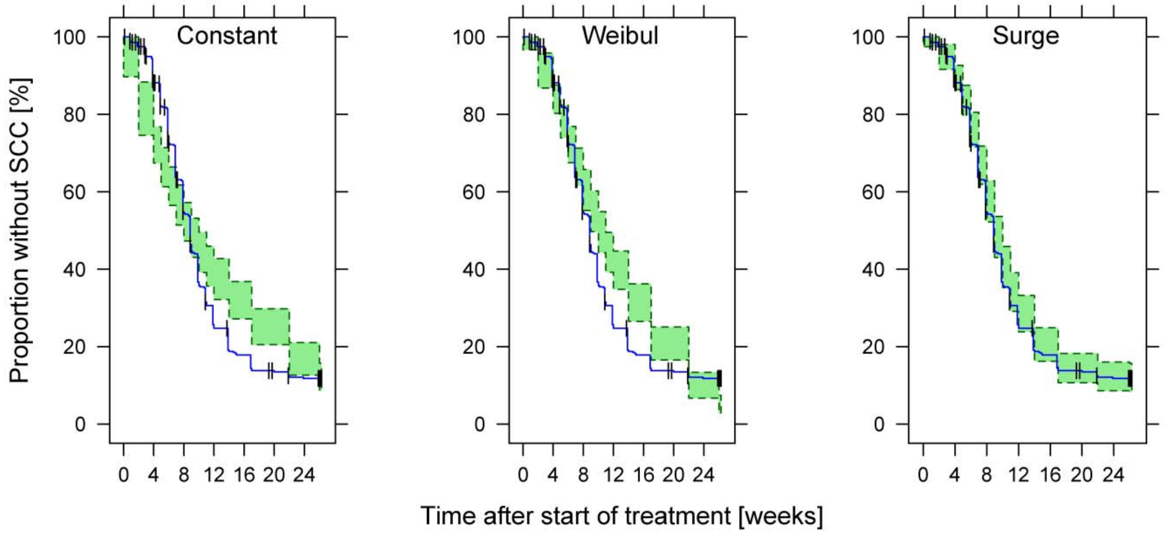

Figure S3. Visual predictive check of evaluated base hazard distributions in the time-to-event model describing TSCC based on liquid cultures, including constant, Weibul and the selected surge function. The solid lines are the Kaplan-Meier curves based on the observed data, vertical tick-marks in the signifies censored data, and the shaded area outlines the 95% prediction interval based on model simulations.

Covariates included in the final model were baseline time to positivity, proportion unavailable culture results, rifampicin exposure, and substitution of ethambutol with moxifloxacin or SQ109. Gender, country, study site, x-ray scoring, presence of cavitation, and pyrazinamide exposure were all significant in univariate analysis, but not in multivariate analysis and therefore not included in the final model. Body weight and isoniazid exposure were not significant at all.

The parameters of the final model were defined according to the equations following below. SA is the surge amplitude, PT is the peak time, SW is the surge width, p denotes the population value and i the individual value. The covariates are denoted with P_{miss} for the proportion unavailable culture results, B_{TTP} for baseline time to positivity, RIF_{AUC} for rifampicin exposure quantified by AUC_{0-24h} (imputed for patients with missing PK data), and MX and SQ for having ethambutol replaced with moxifloxacin or SQ109, respectively (categorical covariates with value 1 if yes, 0 otherwise). The estimated coefficients for the respective relationships are denoted with θ and the covariate name.

$$SA_i = SA_p * \left(\frac{P_{miss,i}}{100}\right)^{\Theta P_{miss}} * \left(\frac{B_{TTP,i}}{4.4}\right)^{\Theta B_{TTP}} * \left(1 + \Theta RIF_{AUC} * \left(\frac{RIF_{AUC,i} - 56.1}{56.1}\right)\right) * (1 + \Theta MX * MX_i) * (1 + \Theta SQ * SQ_i)$$

$$PT_i = PT_p * \left(\frac{B_{TTP,i}}{4.4}\right)^{-\Theta B_{TTP}} * \left(1 - \Theta RIF_{AUC} * \left(\frac{RIF_{AUC,i} - 56.1}{56.1}\right)\right) * (1 - \Theta MX * MX_i) * (1 - \Theta SQ * SQ_i)$$

$$SW_i = SW_p * \left(\frac{B_{TTP,i}}{4.4}\right)^{\Theta B_{TTP}} * (1 - \Theta MX * MX_i) * (1 - \Theta SQ * SQ_i)$$

The statistical significance of each covariate relationship was demonstrated by the increase in objective function value (OFV, defined as minus two time the logarithm of the likelihood) after univariate deletion from the final model with single imputation of rifampicin exposure (Table S2). Given the study design, having moxifloxacin or SQ109 were mutually exclusive. These covariates were therefore only tested together to avoid biasing the typical estimates for patients without substitution of ethambutol. The final parameter estimates after the multiple imputation procedure, including parameter uncertainty, are listed in Table S3. The parameters were estimated with good precision (relative standard errors <30%), with an exception for the coefficients determining effect of moxifloxacin or SQ109 substitution (relative standard errors ~50%).

Table S2. Statistical testing of parameter-covariate relationships in final model.

**Relationship**	**Model modification**	**ΔOFV**	**Degrees of freedom**	**p-value**
P_{miss} – SA	Deleted	55.5	1	<0.001
B_{TTP} – SA/PT/SW	Deleted	51.3	1	<0.001
RIF_{AUC} – SA/PT	Deleted	10.8	1	0.001
MX/SQ – SA/PT/SW	Deleted	12.4	2	0.002
PZA_{AUC} – SA*	Added	-2.96	1	0.09

* Additional evaluation of influence of individual pyrazinamide (PZA) exposure. Other combinations of relationships including PZA C_{max} instead of AUC were also tested, the reported relationship had the largest drop in objective function value (OFV).

Table S3. Final parameter estimates from multiple imputation procedure for the model of TSCC based on liquid cultures.

**Parameters***	**Estimate**	**Uncertainty (relative standard error, %)**
SA_p [day^{-1}]	0.0540	12
PT_p [day]	78.3	4.1
SW_p [day]	35.4	7.0
θP_{miss}	2.06	14
θB_{TTP}	0.211	17
θRIF_{AUC} [%]	3.98	27
θMX [%]	10.0	56
θSQ [%]	-13.6	46

* For definition of abbreviations, see first paragraph in the section Results TTSC liquid cultures.

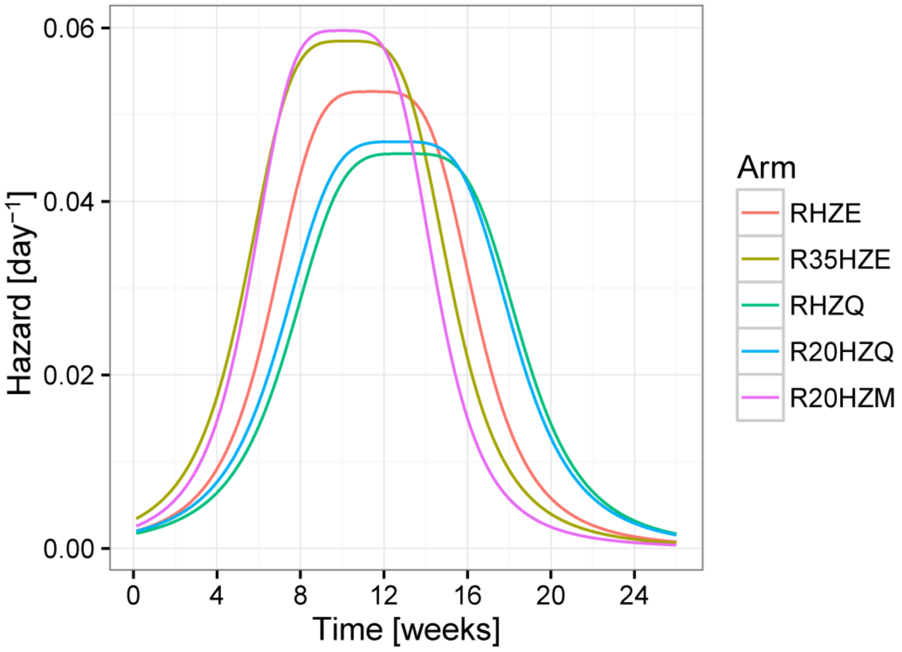

Figure S4. Surge hazard function per arm over time after start of treatment (RHEZ – control, R35HZE – Experimental 1, R35HZE – Experimental 1, RHZQ – Experimental 2, R20HZQ – Experimental 3, R20HZM – Experimental 4)

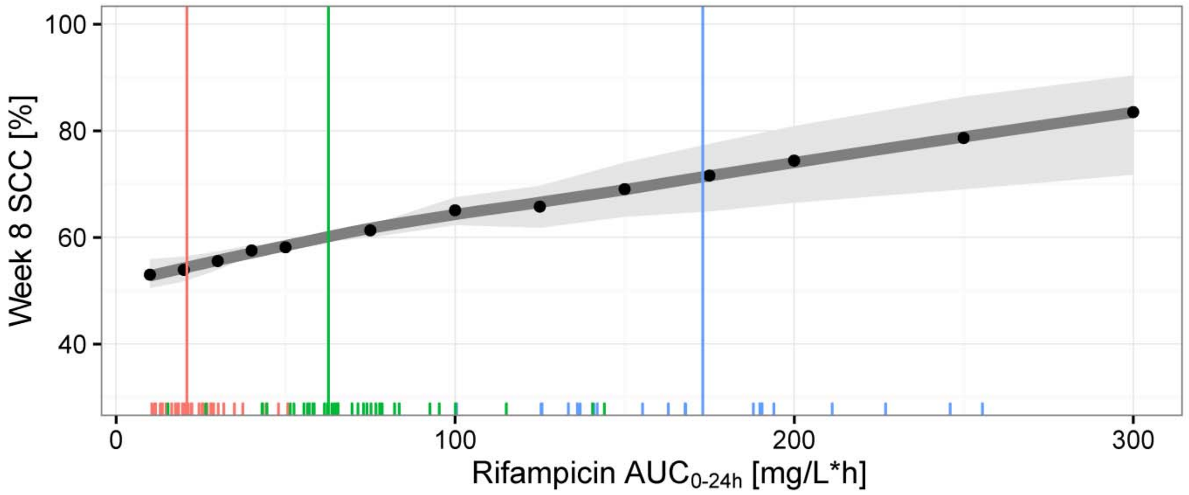

Figure S5. Expected proportion of patients with sputum culture conversion at week eight (Week 8 SCC) over varying rifampicin exposures for a virtual population of patients (distribution of baseline bacterial load mimicking that of the study, no missing culture results) treated with standard doses of isoniazid, pyrazinamide and ethambutol. Black dots are simulation results (n=10,000 in each), the dark grey line is a locally weighted smooth of the simulation results, the light gray shaded area represents a 95% confidence interval based on the uncertainty in the estimate of the parameter for rifampicin effect. Vertical lines represents median observed exposure in the dose groups 10 (red), 20 (green) and 35 (blue) mg/kg, respectively, and the tick marks at the bottom of the graph are individual observed exposures.

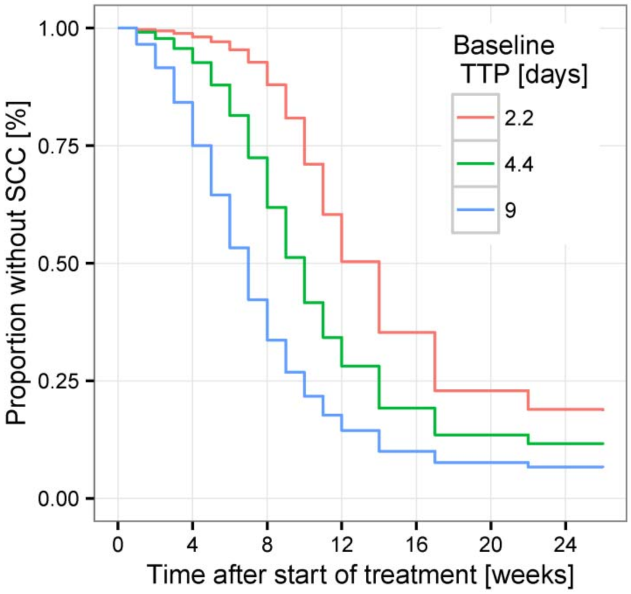

Figure S6. Expected Kaplan-Meier curves (TSCC liquid cultures) for a virtual populations of patients (missing sputum samples mimicking that of the study) treated with ethambutol-containing regimens and having rifampicin $AUC_{0\text{-}24h}$ of 21 mg/L*h (median observed exposure with 10 mg/kg) and having a baseline bacterial load (TTP) of 2.2, 4.4 or 9.0 days (5th, 50th and 95th percentile of observed baseline TTP). *Nota bene*, a short TTP signifies a high baseline bacterial load.

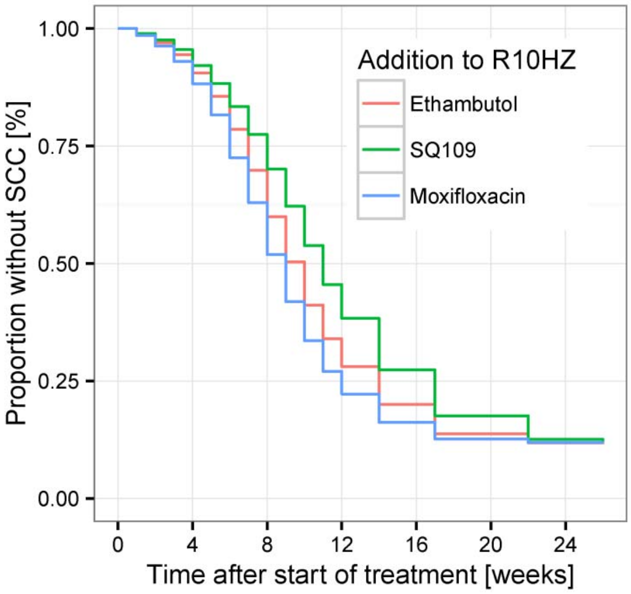

Figure S7. Expected Kaplan-Meier curves (TSCC liquid cultures) for a virtual populations of patients (missing sputum samples and baseline bacterial load mimicking that of the study) having rifampicin $AUC_{0\text{-}24h}$ of 21 mg/L*h (median observed exposure with 10 mg/kg) and as a fourth drug either ethambutol, SQ109 or moxifloxacin.

Results TSCC solid cultures

The best base hazard model for TSCC derived from solid cultures was a Weibull defined by a base and a shape parameter. The covariates found to have a significant impact on the base parameter were baseline bacterial load (power relation, relative to median observed baseline TTP) and rifampicin exposure (linear relation, same centering applied as in the model for liquid cultures, see equations above). The final parameters with uncertainty are listed in Table S4. The model fit is demonstrated in Figure S8.

Table S4. Final parameter estimates from multiple imputation procedure for the model of TSCC based on solid cultures.

**Parameters***	**Estimate**	**Uncertainty (relative standard error, %)**
$BASE_p$ [day^{-1}]	0.0261	3.6
SHAPE	1.74	4.4
θB_{TTP}	0.545	14
θRIF_{AUC} [%]	9.46	40

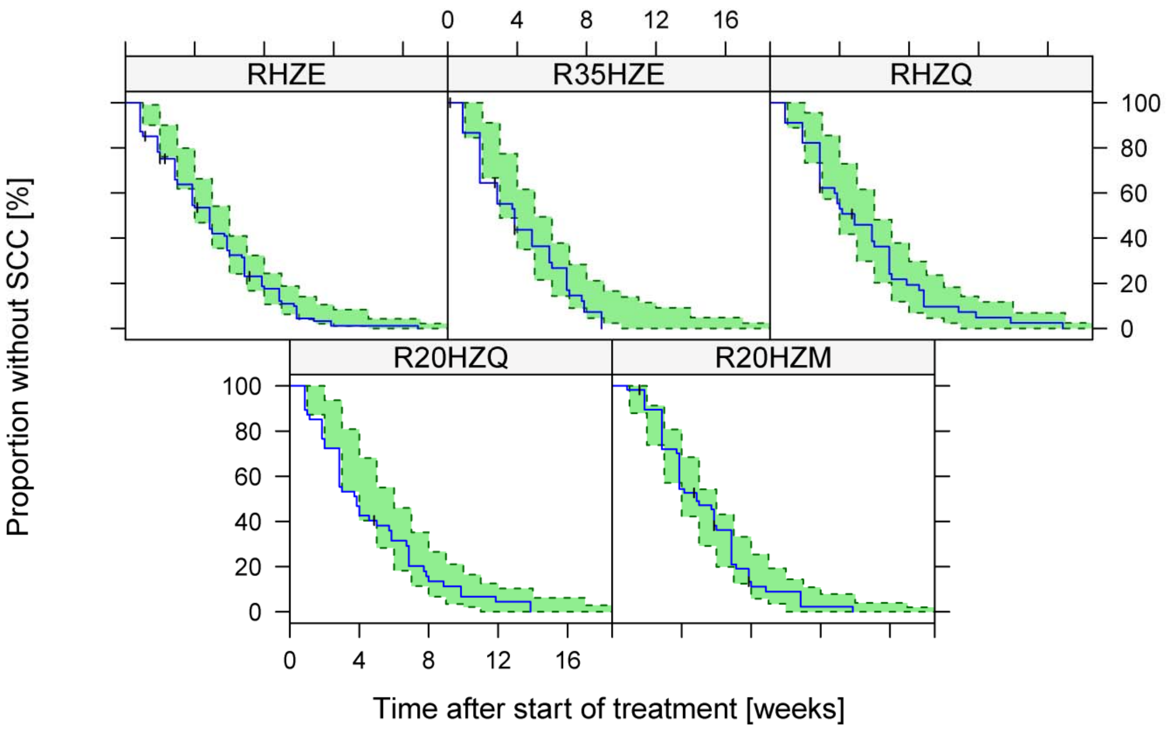

Figure S8. Visual predictive check of the final time-to-event model describing TSCC based on solid cultures, per study arm. The solid lines are the Kaplan-Meier curves based on the observed data, vertical tick-marks signifies censored data, and the shaded area outlines the 95% prediction interval based on model simulations. The arms are control (RHZE) and experimental 1 (R35HZE), experimental 2 (RHZQ), experimental 3 (R20HZQ) and experimental 4 (R20HZM).

NONMEM control streams

Final model TSCC from liquid cultures

```
; Elin Svensson, 2017
; Code for NONMEM 7.3

$PROBLEM    MAMS TSCC liquid
$INPUT      ...

;Sim_start : add/remove for simulation using PsN command "flip_comments"
$DATA     data.csv   IGNORE=@   IGNORE(TYPE.EQ.0)  ; TYPE=0, records for estimation
;$DATA    data.csv   IGNORE=@   IGNORE(TYPE.EQ.1)  ; TYPE=1, records for simulation
;Sim_end

$SUBROUTINE ADVAN=6 TOL=6
$MODEL     COMP=(HAZARD)
$PK

; Define covariates and impute when missing (ARM, MTTP,  AUCR and MISS defined in $IMPUT)

SQ = 0
IF(ARM.EQ.2.OR.ARM.EQ.3) SQ = 1
MOXI = 0
IF(ARM.EQ.4) MOXI = 1
BMTTP = MTTP
IF(MTTP.EQ.-99) BMTTP = 4.365
RIFPK = AUCR
IF(AUCR.EQ.-99) RIFPK = AUCSIM
PSAMP = (100-MISS)

; Define parameters

SA =    THETA(1)*(PSAMP/100)**THETA(7)*(1-THETA(5)*(RIFPK-
56.07486)/56.07486)*(BMTTP/4.365)**THETA(4)*(1 + THETA(6)*MOXI)*(1 -
THETA(8)*SQ)*EXP(ETA(1)) ;the ETA is a placeholder here

PT =     THETA(2)*(1+THETA(5)*(RIFPK-56.07486)/56.07486)*(BMTTP/4.365)**(-THETA(4))*(1 -
THETA(6)*MOXI)*(1 + THETA(8)*SQ)

SW =    THETA(3)*(BMTTP/4.365)**THETA(4)*(1 - THETA(6)*MOXI)*(1 + THETA(8)*SQ)


$DES

DADT(1)= SA / (((T - PT) / SW)**4 + 1)   ;hazard
```

```
$ERROR

;----------TTE Model------------------------------

CHZ = A(1)        ;cumulative hazard
SUR = EXP(-CHZ)   ;survival probability
HAZNOW= SA / (((TIME - PT) / SW)**4 + 1)     ;hazard

IF(DV.EQ.0)   Y=SUR         ;censored event (probability of survival)
IF(DV.NE.0)   Y=SUR*HAZNOW  ;probability density function of event

;----------Simulation Model------------------------------

IF(ICALL.EQ.4) THEN

; For new ID
IF(NEWIND.NE.2) THEN
DV=0
RTTE = 0
ORTTE = 0
CALL RANDOM(2,R)                  ; 2nd distribution (uniform)
USUR=R
ENDIF

; If there was no previous event (SCC or dropout) AND the random
; variable is greater than the probability of survival -> event (SSC)

IF(ORTTE.EQ.0.AND.USUR.GT.SUR) THEN
DV=1
RTTE = 1
ORTTE = 1
ENDIF

; If there was no previous event (SCC or dropout) AND it is the last record -> censoring

IF(ORTTE.EQ.0.AND.LASTR.EQ.1) THEN
DV=0
RTTE = 1
ORTTE = 1
ENDIF

ENDIF

$THETA  …
$OMEGA  0  FIX

;Sim_start : add/remove for simulation using PsN command “flip_comments”
;$SIMULATION (5988566) (39978 UNIFORM) ONLYSIM NOPREDICTION
$ESTIMATION MAXEVAL=9999 METHOD=0 LIKE SIGL=6 NSIG=2 MSFO=msfb210
;Sim_end
```

Final model TSCC from solid cultures

```
; Elin Svensson, 2017
; Code for NONMEM 7.3

$PROBLEM    MAMS TSCC liquid
$INPUT      …

;Sim_start : add/remove for simulation using PsN command "flip_comments"
$DATA      data.csv   IGNORE=@   IGNORE(TYPE.EQ.0)  ; TYPE=0, records for estimation
;$DATA     data.csv   IGNORE=@   IGNORE(TYPE.EQ.1)  ; TYPE=1, records for simulation
;Sim_end

$SUBROUTINE ADVAN=6 TOL=6
$MODEL      COMP=(HAZARD)
$PK

; Define covariates and impute when missing (MTTP and  AUCR defined in $IMPUT)

BMTTP = MTTP
IF(MTTP.EQ.-99) BMTTP = 4.365
RIFPK = AUCR
IF(AUCR.EQ.-99) RIFPK = AUCSIM

; Define parameters

BASE = THETA(1)*(BMTTP/4.365)**THETA(3)*(1+THETA(4)*(RIFPK-56.07486)/56.07486)*EXP(ETA(1))
;the ETA is a placeholder here
SHP = THETA(2)
DEL=1E-6


$DES

DADT(1)=BASE*SHP*(BASE*(T+DEL))**(SHP-1)   ;


$ERROR

;----------TTE Model------------------------------

CHZ = A(1)        ;cumulative hazard
SUR = EXP(-CHZ)   ;survival probability
HAZNOW=BASE*SHP*(BASE*(TIME+DEL))**(SHP-1)    ;hazard
IF(DV.EQ.0)   Y=SUR         ;censored event (probability of survival)
IF(DV.NE.0)   Y=SUR*HAZNOW  ;probability density function of event
```

```
;----------Simulation Model------------------------------

IF(ICALL.EQ.4) THEN

; For new ID

IF(NEWIND.NE.2) THEN
DV=0
RTTE = 0
ORTTE = 0
CALL RANDOM(2,R)                   ; 2nd distribution (uniform)
USUR=R
ENDIF

; If there was no previous event (SCC or dropout) AND the random
; variable is greater than the proba of survival -> event (SSC)

IF(ORTTE.EQ.0.AND.USUR.GT.SUR) THEN
DV=1
RTTE = 1
ORTTE = 1
ENDIF

; If there was no previous event (SCC or dropout) AND it is the last record -> censoring

IF(ORTTE.EQ.0.AND.LASTR.EQ.1) THEN
DV=0
RTTE = 1
ORTTE = 1
ENDIF

ENDIF



$THETA  …

$OMEGA  0  FIX

;Sim_start : add/remove for simulation
;$SIMULATION (5988566) (39978 UNIFORM) ONLYSIM NOPREDICTION
$ESTIMATION MAXEVAL=9999 METHOD=0 LIKE SIGL=6 NSIG=2
;Sim_end
```
